# Safety and effectiveness of Evicel^®^ fibrin sealant as an adjunct to sutured dural repair in children undergoing cranial neurosurgery

**DOI:** 10.1007/s00381-024-06434-4

**Published:** 2024-05-10

**Authors:** Gnanamurthy Sivakumar, Shailendra Magdum, Kristian Aquilina, Jothy Kandasamy, Vivek Josan, Bogdan Ilie, Ellie Barnett, Richard Kocharian, Benedetta Pettorini

**Affiliations:** 1 Leeds General Infirmary, Great George Street, Leeds, LS1 3EX UK; 2 John Radcliffe Hospital, Headley Way, Headington, Oxford, OX3 9DU UK; 3 Great Ormond Street Hospital, London, WC1N 3JH, UK; 4 Royal Hospital for Sick Children, Sciennes Road, Edinburgh, EH9 1LF UK; 5 Royal Manchester Children’s Hospital, Oxford Road, Manchester, M13 9WL UK; 6 Ethicon, Inc., 1000 US-202 South, Raritan, NJ 08869 USA; 7 Ethicon, Inc., 8 Deer Park, Livingston, EH54 8AF United Kingdom; 8 Alder Hey Children’s Hospital, Eaton Road, Liverpool, L12 2AP UK

**Keywords:** Dural sealing, Pediatric neurosurgery, Fibrin sealant, Cerebrospinal fluid leak

## Abstract

**Purpose:**

Cerebrospinal fluid (CSF) leakage is a challenging complication of intradural cranial surgery, and children are particularly at risk. The use of dural sealants confers protection in adults, but pediatric studies are scarce. We evaluated the safety and efficacy of Evicel^®^ fibrin sealant as an adjunct to primary dural suturing in children undergoing cranial surgery.

**Methods:**

A multicenter trial prospectively enrolled pediatric subjects (< 18 years) undergoing cranial neurosurgery who, upon completion of primary sutured dural repair, experienced CSF leakage. As agreed by the EMA Evicel^®^ Pediatric Investigation Plan, 40 subjects were intra-operatively randomized 2:1 to Evicel^®^ or additional sutures (‘Sutures’). Data analysis was descriptive. The efficacy endpoint was treatment success rate, with success defined as intra-operative watertight closure after provocative Valsalva maneuver (primary endpoint). Safety endpoints were postoperative CSF leakage (incisional CSF leakage, pseudomeningocele or both) and surgical site complications (secondary endpoints).

**Results:**

Forty subjects (0.6–17 years) were randomized to Evicel^®^ (N = 25) or Sutures (N = 15) (intention-to-treat). Intracranial tumor was the most common indication and procedures were mostly supratentorial craniotomies. Success rates were 92.0% for Evicel^®^ and 33.3% for Sutures, with a 2.76 estimated ratio of success rates (Farrington-Manning 95% CI [1.53, 6.16]). Sensitivity analyses in per-protocol and safety sets showed similar results. Despite a higher rescue treatment rate, the frequencies of postoperative CSF leakage and wound complications were higher for Sutures than for Evicel^®^.

**Conclusion:**

This small-scale prospective study shows Evicel^®^ treatment to be safe and effective as an adjunct to primary sutured dura mater closure in a pediatric population. Compared to additional sutures, Evicel^®^ was associated with reduced postoperative CSF leakage and surgical site complications. (Trial registration: The trial was registered as NCT02309645 and EudraCT 2013-003558-26).

**Supplementary Information:**

The online version contains supplementary material available at 10.1007/s00381-024-06434-4.

## Background

Postoperative leakage of cerebrospinal fluid (CSF) is a challenging complication of intradural cranial surgery, as it may interfere with wound healing and lead to pseudomeningocele, surgical site infection, meningitis and fistula. Risk factors for CSF leakage include younger age and infratentorial or intraventricular approaches [1–3]. These place children at an increased risk, but because pediatric studies are heterogeneous and often of low quality, the incidence and risk profiles in children remain incompletely understood. Interpretation of published rates, which vary between 0 and 38%, is further hindered by the variable use of adjunctive and rescue treatment, and by inconsistency in the definitions for CSF leakage [3, 4]. A meta-analysis of good-quality studies estimated the overall incidence at 7.4% [3], concurring with a more recent large retrospective study [4]. Aside from causing patient morbidity, CSF leakage and its complications lead to significant health care expenditure [5, 6]. Pediatric series have reported rates of invasive measures of 49.1% and 70.0% [3, 7].

According to the longstanding tenet in neurosurgery, the most important measure to prevent CSF leakage is a meticulous repair of the anatomical dural barrier [8]. Techniques include primary and additional suturing of the native dura, adjunctive use of dural sealants, and, in case of dural defect, dural graft augmentation with biologic or synthetic material. In a recent retrospective multicenter study of 2,310 subjects, multivariate regression analysis showed dural sealant use to be associated with reduced incisional CSF leakage and reduced wound infection [1], and this is supported by a body of prior randomized and non-randomized adult studies [9–19]. Duraplasty was associated with an increased risk of incisional CSF leak [1]. However, the authors of the study caution that this result may be confounded by the large variability in dural substitutes, because individual studies have provided evidence to the contrary [20, 21]. Because pediatric series are limited and retrospective, the effects of dural sealants in children are difficult to estimate [3, 4], and those of duraplasty have been inconsistent [3, 4, 20, 21].

High-level evidence for the value of dural sealants was derived from a randomized controlled trial (RCT) which demonstrated that Evicel^®^, a human-derived fibrin sealant, was superior to additional suturing in providing intra-operative watertight dura mater closure [9]. More recently, a RCT confirmed this for BioSeal^®^, a porcine-derived fibrin sealant [22]. Originally developed as topical hemostats, fibrin sealants are mainly composed of thrombin and fibrinogen [14, 23]. Their safety and hemostatic efficacy were demonstrated in clinical trials of several surgical disciplines [24–27], supporting their clinical indication in the United States (US) and the European Union (EU) [28, 29]. As adjuncts to dura repair [14, 23], fibrin sealants present the advantages over synthetic sealants that they do not swell and thus avoid causing additional intracranial pressure, and that they resorb physiologically through fibrinolysis and thus do not lead to foreign body reactions. In the EU, Evicel^®^ is indicated for suture line sealing in dura mater closure in adults [28, 29].

To address the need for effective measures to prevent CSF leakage in pediatric neurosurgery, we conducted a RCT evaluating Evicel^®^ fibrin sealant versus additional sutures, as adjunct treatments to primary sutured dural closure in children undergoing intradural cranial surgery. The study aimed to evaluate the safety and efficacy of Evicel^®^ as an adjunct to primary sutures to obtain intra-operative watertight closure.

## Methods

### Trial design

This prospective, open-label, multicenter, randomized controlled clinical trial evaluated the safety and effectiveness of Evicel^®^ as an adjunct to sutured dural repair in children undergoing cranial neurosurgery, in comparison to additional suturing. The trial was conducted at 7 institutions in the United Kingdom (UK). Ethical considerations prevented the use of a sham control group and required that rescue treatment be provided when study treatment fails to achieve dural closure. The design was approved by the European Medicines Agency (EMA) as part of a Paediatric Investigation Plan (PIP) for Evicel^®^ (EMEA-001149-PIP01-11-M07), and aligned with EU Guidance on clinical investigation of plasma-derived fibrin sealants (CPMP/BPWG/1089/00, 29 July 2004) (control group receiving treatment without fibrin sealant, that is approved for the pediatric indication, and surgical situations representing regular clinical practice). The design described in the PIP required a minimum of 40 subjects to be randomized in a 2:1 ratio to Evicel^®^ or additional suturing (‘Sutures’), with stratification according to infra- and supratentorial approach. The trial was conducted in accordance with the International Conference on Harmonization (ICH) Harmonized Tripartite Guideline for Good Clinical Practice (2016); the Declaration of Helsinki (2013); the European Union Clinical Trial Directive (2001/20/EC, May 2001); and the EU GCP Directive (2005/28/EC). Approval from the Office for Research Ethics Committees Northern Ireland, Lisburn, UK, was obtained prior to commencing the trial. Original clinical trial application approval was obtained from UK MHRA on 12 June 2014. The trial was registered as NCT02309645 and EudraCT 2013-003558-26.

### Study subjects and procedure

Subjects younger than 18 years requiring elective or emergent craniotomy or craniectomy for an infra- or supratentorial procedure were considered. Per local regulations, the subject’s parent or legal representative provided informed consent; assent was obtained from subjects who possessed the intellectual and emotional ability to comprehend the trial’s concepts. Patients were enrolled intra-operatively if -upon completion of primary sutured dural repair- a CSF leak was apparent (spontaneously or after Valsalva maneuver) in a Class I surgical wound with dural cuffs sufficiently wide to allow for coverage by fibrin sealant or placement of additional sutures. Surgeons used their institution’s standard clinical practice for the type and technique of primary suture closure of the dura. Exclusion criteria were existing surgical dural lesions with potential CSF leakage, hydrocephalus, existing CSF drainage or burr holes with damage to the dura, or known hypersensitivity to Evicel^®^ component(s). Inclusion and exclusion criteria are fully listed in Supplementary Information 1.

Upon enrollment, subjects were randomized in a 2:1 ratio to Evicel^®^ or Sutures. The Sponsor provided 2 sets (supra- and infratentorial) of computer-generated randomization envelopes containing randomization number and treatment allocation. For Evicel^®^-randomized subjects, 1 or 2 thin layers were applied (1- to 2-min cure time between layers), covering the suture line and ≥5 mm adjacent area including all suture holes. Evicel^®^ was applied by drip or spray using the applicator device and accessory tips, according to surgeon preference [28]. The amount depended on the suture area and application method, with no maximum dose. If a second Valsalva maneuver showed persistent CSF leakage, a second treatment (1 or 2 layers) could be applied. If a subject was randomized to Sutures, additional suturing was performed per the investigator’s institutional standard of care.

Following randomized treatment, if final provocative Valsalva maneuver showed watertight closure, the treatment was deemed successful. For Evicel^®^-randomized subjects, no further adjunct was allowed. For Sutures-randomized subjects, adjunctive treatments (except fibrin sealants) were allowed to assure closure durability. If, after final Valsalva, CSF leakage was still apparent, the subject was deemed a treatment failure. For rescue treatment, the surgeon was to revert to their institutional standard of care for closure, which could include on-lay dural patch or fibrin sealants (except Evicel^®^). Post-operatively, subjects were followed until hospital discharge and, on post-operative days 5 (±2) and 30 (±3), with an office visit.

### Primary endpoint

The primary endpoint related to efficacy. It was defined as the proportion of subjects with treatment success, considered as the achievement of intra-operative watertight closure after completion of the randomized treatment as assessed by provocative testing with Valsalva maneuver.

### Secondary endpoints

Secondary endpoints related to safety. Adverse events (AE) and serious adverse events (SAE) definitions complied with the EU Clinical Trial Directive, [30, 31] were collected from randomization until postoperative day 30 (±3) and were adjudicated for relationship with study product (Evicel^®^ arm only, as no study product was used in the Sutures arm) and procedure. Specific safety endpoints were the incidence of postoperative CSF leakage, and surgical site complications including infection, seroma and hematoma [32]. Under postoperative CSF leak, a distinction was made between incisional CSF (iCSF) leak, pseudomeningocele, or a combination of both. The iCSF leak and pseudomeningocele were defined by clinical observation, diagnostic testing, or the need for surgical intervention. Because they were considered important medical events (as potential treatment failures), any events of postoperative CSF leakage were to be categorized as SAEs.

### Statistical analysis

The proportions of treatment success were summarized descriptively by treatment group, including the estimated ratio of success proportions with 2-sided 95% confidence interval (CI) (Farrington-Manning). The primary analysis was based on the intention-to-treat (ITT) set and included summaries of success proportions within the infra- and supratentorial stratum. Sensitivity analyses were performed in the Per-protocol (PP) and Safety sets. Safety endpoints were summarized descriptively. Software SAS^®^ Version 9.1 [EG] was used.

## Results

### Study subjects

**Fig. 1:**
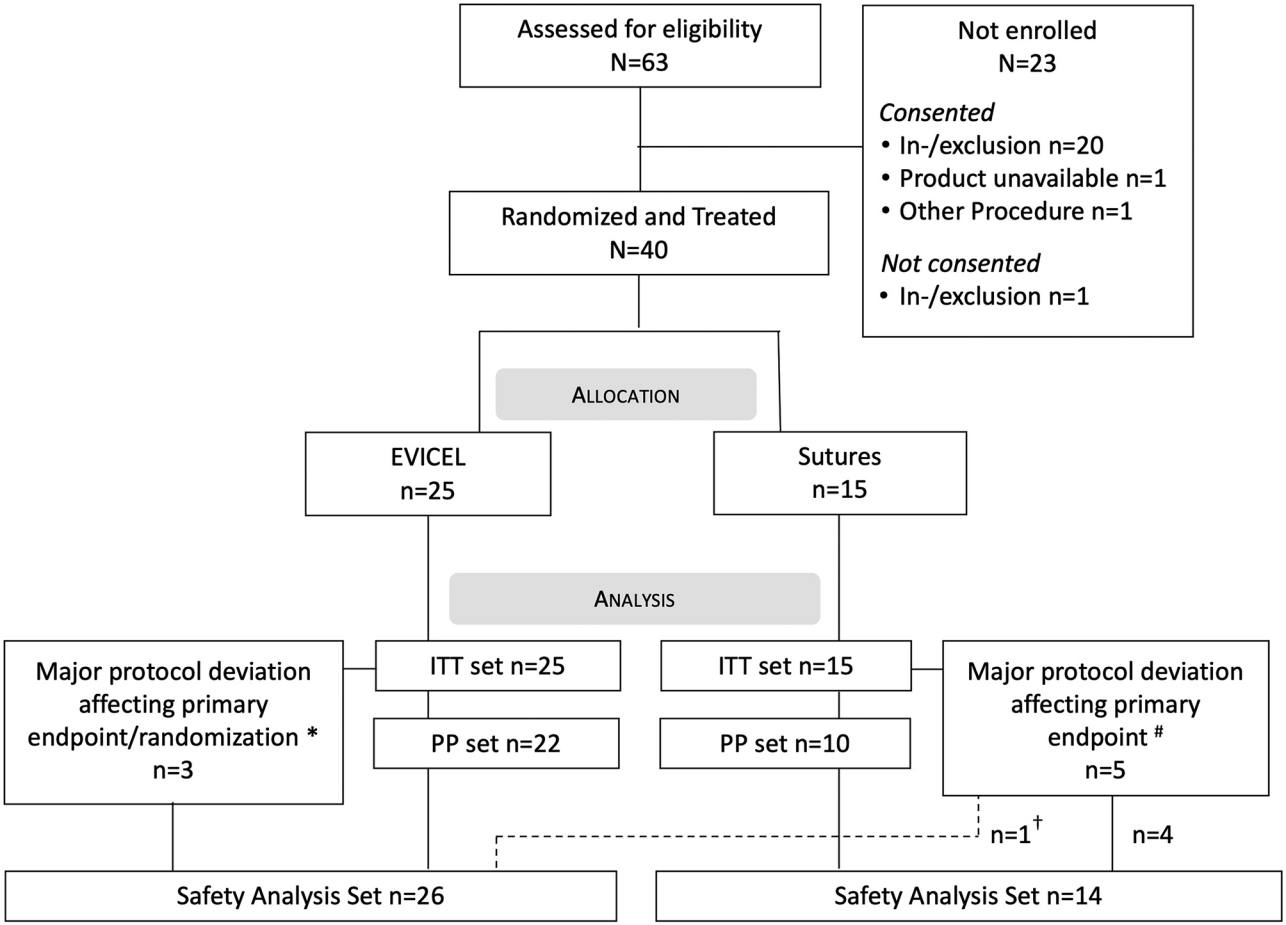
Consort flow diagram with disposition of study subjects. Shown are the Intention-to-treat set (ITT), Per protocol set (PP) and Safety set. * Exclusions due to existing endoscopic third ventriculostomy (n = 1), ventriculoperitoneal drain (n = 1), and external ventricular drain (n = 1). ^#^ Exclusions due to misrandomization (n = 2), procedure deviation (Valsalva not done after additional sutures) (n = 1), existing ventriculoperitoneal drain (n = 1) and external ventricular drain (n = 1). ^†^ Subject received Evicel^®^ instead of Sutures and is therefore included in Evicel^®^ Safety Analysis Set

Between 9 October 2014 and 17 September 2021, 40 of 63 screened subjects were randomized to Evicel^®^ (‘Evicel’) (N = 25) or Sutures (N = 15) (ITT set) (Fig. 1). The baseline patient characteristics (Table 1) were similar across groups. There were 8 major protocol deviations affecting randomization or primary endpoint (3 Evicel^®^ and 5 Sutures). The PP set comprised 22 Evicel^®^ and 10 Sutures subjects. Five major protocol deviations (3 Evicel^®^ and 2 Sutures) occurred at one site and concerned the exclusion criteria ‘existing CSF drainage’ (Fig. 1). These were reported to MHRA under the serious breach category and reviewed by the trial’s safety lead and biostatistician. All subject data were re-monitored. It was confirmed that the safety of enrolled subjects had not been compromised, and a sensitivity analysis was performed on the PP set. The Safety set counted 26 Evicel^®^ and 14 Sutures subjects because 1 Sutures-allocated subject received Evicel^®^ (major protocol deviation). This subject was included under Sutures for the ITT analysis and included under Evicel^®^ for sensitivity analysis in the Safety set.

**Table 1:** Patient characteristics (ITT Set). Medical history shows disorders that occurred in ≥ 10% of subjects

	**Evicel**^**®**^** (N = 25)**	**Sutures (N = 15)**
Age (years), median (range)	10.0 (0.8,17.0)	10.0 (0.6,15.0)
Male/Female ratio, n (%)/n (%)	14 (56.0%) / 11 (44.0%)	9 (60.0%) / 6 (40.0%)
BMI (kg/m^2^), median (range) ^a^	18.1 (13.4,33.8)	18.5 (14.0,23.7)
Medical History, n (%)		
Any previous surgery	8 (32.0%)	4 (26.7%)
Neoplasms	16 (64.0%)	11 (73.3%)
Nervous system disorders	14 (56.0%)	12 (80.0%)
Congenital/genetic disorders	9 (36.0%)	4 (26.7%)
Immune disorders	4 (16.0%)	0
Cardiac disorders	2 (8.0%)	2 (13.3%)
Infectious disorders	4 (16.0%)	0

^a^Evicel^®^ n = 15, Sutures n = 10

### Surgical procedures and operative parameters

In both groups, most procedures were craniotomies and supratentorial approaches (Table 2, ITT set); the most frequent surgical indication was intracranial tumor, and more than half of intra-operative CSF leaks were spontaneous. The surgical and operation room times were similar between groups; hospitalization time was slightly longer for Sutures than for Evicel^®^. All but 3 Evicel^®^-treated subjects received 1 treatment, mostly of 1 product layer (Table 3). In Sutures-treated subjects, the median number of sutures was low (2.0), albeit with a wide range (1.0–12.0); 1 subject received additional treatment for suture durability. After completion of study treatment, 3 Evicel^®^-treated subjects (11.5%) and 9 Sutures-treated subjects (64.3%) received rescue treatment for persistent CSF leak. The quantitative use of Evicel^®^ product is described in Supplementary Information 2.

**Table 2:** Procedural characteristics (ITT Set)

	**Evicel**^**®**^** (N = 25)**	**Sutures (N = 15)**
**Operative procedure**		
Procedure type, n subjects (% of total N)		
Craniotomy	24 (96.0%)	15 (100.0%)
Craniectomy	1 (4.0%)	0 (0.0%)
Approach, n subjects (% of total N)		
Infratentorial	4 (16.0%)	3 (20.0%)
Intracranial tumor	3 (12.0%)	3 (20.0%)
Chiari malformation	1 (4.0%)	0
Supratentorial	21 (84.0%)	12 (80.0%)
Intracranial tumor	13 (52.0%)	9 (60.0%)
Epilepsy	6 (24.0%)	1 (6.7%)
A-V malformation	2 (8.0%)	1 (6.7%)
Arachnoid cyst	0	1 (6.7%)
CSF leak before randomization, n subjects (% of total N)		
Spontaneous	14 (56.0%)	10 (66.7%)
After Valsalva Maneuver	11 (44.0%)	5 (33.3%)
Duration of surgery, min, median (range)^a^	305 (123, 452)	288 (188, 675)
Time in operation room, min, median (range)^a^	376 (160, 561)	361 (214, 778)
Postoperative hospital stay, nights, median (range)	5 (2, 25)	7 (2, 35)

^a^Evicel^®^ n = 23, Sutures n = 14

**Table 3:** Treatment parameters and intra-operative outcomes (safety set)

**Evicel**^®^			**N = 26**
Number of layers within each treatment, n subjects (% of total N = 26)			
* 1st Treatment*	1 layer		18 (69.2%)
	2 layers		8 (30.8%)
* 2nd Treatment*	1 layer		1 (3.9%)
	2 layers		2 (7.7%)
Intra-operative outcome following each treatment, n subjects (% of total N = 26)			
* 1st Treatment*	No CSF Leak		23 (88.5%)
	CSF Leak		3 (11.5%)
* 2nd Treatment*	No CSF Leak		0
	CSF Leak		3 (11.5%)
Rescue treatment, n subjects (%)			3 (11.5%)^a^

**Sutures**			**N = 14**
Number of additional sutures, median (range)			2.0 (1.0,12.0)
Additional treatment for durability, n subjects (%)			1 (7.1%)^b^
Intra-operative outcome, n subjects (% of total N)			
	No CSF leak		5 (35.7%)
	CSF leak		9 (64.3%)
Rescue treatment, n subjects (% of total N)			9 (64.3%)^c^

^a^Infratentorial: DuraSeal^®^ n = 1, Surgicel^®^ + Duragen^®^ + Duraguard^®^ n = 1. Supratentorial: Tisseel^®^ + Surgicel^®^ n = 1

^b^Surgicel^®^ Fibrillar

^c^Infratentorial: Pericranium n = 1, Adherus^®^ + Duraguard^®^ n = 1. Supratentorial: Tisseel^®^ n = 2, Tisseel^®^ + Surgicel^®^ n = 1, Tisseel^®^ + Duragen^®^ n = 1, Tisseel^®^ + Surgicel^®^ + fascia n = 1, Surgicel^®^ + Gelfoam^®^ n = 1, Gelfoam^®^ + Spongostan^®^ n = 1

### Primary endpoint: efficacy

In the ITT analysis, success rates were 92.0% (23/25) for Evicel^®^ and 33.3% (5/15) for Sutures, establishing an estimated P_E_/P_S_ ratio of 2.76 (95% CI: [1.53, 6.16]) (Table 4). Sensitivity analyses in the PP and Safety sets showed similar rate differences: 90.9% (20/22) for Evicel^®^ and 40.0% (4/10) for Sutures with an estimated P_E_/P_S_ ratio of 2.27 (95% CI: [1.27, 5.53]) in the PP set, and 88.5% (23/26) for Evicel and 35.7% (5/14) for Sutures with an estimated ratio P_E_/P_S_ of 2.48 (95% CI: [1.39, 5.52]) in the Safety set. Although subjects undergoing infratentorial surgery were few, analysis by surgical approach (ITT set) showed that, within both the supra- and infratentorial stratum, the success rates were markedly higher for Evicel^®^ than for Sutures, while for both Evicel^®^ and Sutures, success rates were higher for supratentorial than for infratentorial procedures.

**Table 4:** Primary efficacy analysis and sensitivity analyses. Success was defined as the achievement of intra-operative watertight closure after completion of the randomized treatment, as assessed by provocative testing with valsalva maneuver

	**Evicel**^**®**^ **Success Rate** **% (n/N)**	**Sutures** **Success Rate** **% (n/N)**	**Estimated P**_**E**_**/P**_**S**_	**Farrington-Manning** **95% CI for P**_**E**_**/P**_**S**_
***Intention-to-treat set***				
Overall group	92.0% (23/25)	33.3% (5/15)	2.76	(1.53, 6.16)
Infratentorial	50.0% (2/4)	0.0% (0/3)		
Supratentorial	100.0% (21/21)	41.7% (5/12)		

***Per protocol set***				
Overall group	90.9% (20/22)	40.0% (4/10)	2.27	(1.27, 5.53)

***Safety set***				
Overall group	88.5% (23/26)	35.7% (5/14)	2.48	(1.39, 5.52)

P_E_/P_S_, Proportion successes in Evicel^®^ group / Proportion successes in Sutures group

*CI* Confidence Interval

### Secondary endpoints: safety

All Sutures-treated subjects and most Evicel^®^-treated subjects (84.6%) experienced at least 1 AE (Table 5). Seven AEs in 5 Evicel^®^-treated subjects (19.2%) and 16 AEs in 8 Sutures-treated subjects (57.1%) were considered serious (Table 5). One SAE of pseudomeningocele in an Evicel^®^-treated subject was considered related to study product by the Sponsor, while most other SAEs in both groups were considered related or possibly related to the procedure (85.7% and 87.5% for Evicel^®^ and Sutures, respectively) (Table 5).

**Table 5:** Adverse events and relatedness to study product (determined for evicel^®^ group only) and procedure (safety set)

	**Evicel**^**®**^** (N = 26)**	**Sutures (N = 14)**
**Individual AE, n (%)**		
Individual AEs	118	110
Related or possibly related to study product	0	N/A
Related or possibly related to study procedure	71 (60.2%)	73 (66.4%)
Individual SAEs	7	16^a^
Related or possibly related to study product	1 (3.8%)^b^	N/A
Related or possibly related to study procedure	6 (85.7%)^c^	14 (87.5%)^d^

**Subjects with AE, n (%)**		
≥ 1 A*E*	22 (84.6%)	14 (100.0%)
≥ 1 Serious AE	5 (19.2%)	8 (57.1%)
≥ 1 Severe AE	2 (7.7%)	1 (7.1%)
≥ 1 AE related or possibly related to product	0	N/A
≥ 1 SAE related or possibly related to product	1 (3.8%)^b^	N/A
≥ 1 AE related or possibly related to procedure	21 (80.8%)	12 (85.7%)
≥ 1 SAE related or possibly related to procedure	5 (19.2%)	7 (50.0%)

^a^Partial seizure, neurofibromatosis and 14 other SAE as detailed in footnote (d)

^b^Pseudomeningocele (Causality upgraded by Sponsor)

^c^Diabetes insipidus, pyrexia, meningitis, medulloblastoma recurrence, convulsive seizure, hydrocephalus (due to malfunction existing CSF shunt),

^d^Pseudomeningocele with iCSF leak (n = 1), pseudomeningocele without iCSF leak (n = 4), hematoma (n = 2), vomiting (n = 1), hemorrhagic cyst (n = 1), shunt infection (n = 1), pneumocephalus (n = 1), transverse sinus thrombosis (n = 1), hydrocephalus (due to malfunction existing CSF shunt) n = 1, hydrocephalus (due to choroid plexus carcinoma and intraventricular blood collection) n = 1

Events of postoperative CSF leak were categorized as SAEs: 1 pseudomeningocele for Evicel^®^, 1 pseudomeningocele with iCSF leak and 4 pseudomeningoceles without iCSF leak for Sutures (Table 5).

Overall, 38 AEs (16.7%) were categorized as nervous system disorders. These occurred in 13 Evicel^®^-treated (50.0%) and 10 Sutures-treated subjects (71.4%) and included headache (9 Evicel^®^-treated (34.6%) and 5 Sutures-treated (35.7%) subjects), and seizures (1 Evicel^®^-treated (3.8%) and 1 Sutures-treated subject (7.1%)).

Surgical site complications were overall less frequent in Evicel^®^- than in Sutures-treated subjects (Table 6). Specifically, the frequency of pseudomeningocele, and to a lesser degree, those of iCSF leak, infection and hematoma, were lower after treatment with Evicel^®^ than Sutures.

**Table 6:** Surgical site complications observed within 30 days postoperatively (safety set). All complications are also reported as AEs (Table 5)

**Surgical site complications, n subjects (%)**	**Evicel**^**®**^** (N = 26)**	**Sutures (N = 14)**
≥ 1 surgical site complication	9 (34.6%)	8 (57.1%)
CSF leakage	1 (3.8%)	5 (35.7%)
Incisional CSF leakage	0	0
Pseudomeningocele	1 (3.8%)^a^	4 (28.6%)^b^
Pseudomeningocele and incisional CSF leakage	0	1 (7.1%)^c^
Infection	1 (3.8%)	1 (7.1%)
Hematoma	1 (3.8%)	1 (7.1%)
Other	6 (23.1%)^d^	6 (42.9%)^e^

^a^No treatment needed

^b^Treated with CSF shunt (n = 2), no treatment needed (n = 2)

^c^Treated with pressure bandage and re-suturing

^d^Itchy wound, soreness/numbness, hydrocephalus (due to malfunction existing CSF shunt), subgaleal collection, subgaleal swelling, weeping sutures

^e^Hydrocephalus (due to intraventricular blood collection), hydrocephalus (due to malfunction existing CSF shunt), non-occlusive sinus transversus thrombus, wound swelling, pneumocephalus, bruising

## Discussion

This trial supports the safety and efficacy of Evicel^®^ as an adjunct to primary sutured dural repair in children undergoing cranial neurosurgery. The trial was conducted as part of a PIP for Evicel^®^, following approval for this indication in adults. The data mirrors the findings from an earlier clinical trial in adults, by showing that Evicel^®^ performed better than additional sutures in establishing watertight closure of a primary sutured incision, with no safety signals identified [9].

This trial is the first to prospectively investigate fibrin sealant use to prevent CSF leakage in children after cranial neurosurgery, with an intra-operative primary efficacy endpoint and additional suturing as a comparator [9]. The observed 2.76 success rate ratio of Evicel^®^ to Sutures was accompanied by lower rates of postoperative CSF leakage, consisting of iCSF leakage and/or pseudomeningocele, for Evicel^®^, despite a higher rescue treatment rate in the Sutures group. This supports the adage in neurosurgery that intra-operative watertight dural repair protects against postoperative CSF leakage and its complications, and should be aimed for [8]. In addition to a scarcity of data, current literature on the use of fibrin sealants in pediatric patients is exclusively retrospective and shows high variability in study design, type of dural incision, primary treatment, definition of CSF leak and other outcome measures, and duration of follow-up. As suggested by Slot et al., this may explain why their recent meta-analysis and large retrospective series of pediatric populations failed to show a significant effect of watertight closure on the incidence of postoperative CSF leakage [3, 4]. The authors specifically indicated that most studies were of fair-to-low quality and failed to clearly define CSF leak as a (primary) outcome [3, 4]. In accordance with Evicel^®^’s current clinical indication [28, 29], the current trial included only dural incisions that allowed for primary suturing of the dural cuff. When such is not possible, e.g. due to dural defects, today’s advanced neurosurgical techniques and dural substitutes help prevent postoperative complications [3, 20, 21, 33]. Recent studies questioning the true need for watertight closure report mostly on supratentorial procedures (with lower hydrostatic pressure) and dural incisions that were systematically augmented by duraplasty or dural overlay [21, 33, 34]. The current trial supports that in scenarios where sutured closure is possible, fibrin sealants such as Evicel^®^ are an effective, readily available, and non-invasive adjunct to obtain watertight closure [9]. Larger controlled trials are needed to demonstrate cost/effectiveness of the currently available adjunctive treatments for dural closure in the pediatric population.

In addition to lower rates of pseudomeningocele and iCSF leak, surgical site complications such as infection, bleeding and other complications were less frequent following Evicel^®^ than additional suturing. Originally developed as hemostats, fibrin sealants may help hemostasis and were shown in multiple randomized and non-randomized studies to support the prevention of surgical site infections [9–19]. Additional sutures, in contrast, may inflict further dural damage through needle holes and dural traction. As a potential treatment failure, the single pseudomeningocele in the Evicel^®^ group was considered an SAE possibly related to Evicel^®^ product. The Sutures group counted 5 SAE of pseudomeningocele, but causal relatedness was not part of the trial’s safety evaluation since no adjunct product was used in that group. Comorbidities were highly prevalent in the study population; these related mostly to serious conditions underlying the surgical indications and, together with the major cranial procedure, may help explain why most subjects experienced one or more AEs. Expectedly, the incidence of AEs was similar in the two groups, as most AEs were considered related or possibly related to the procedure. For Evicel^®^, however, no new safety signals were identified. In contrast to its predecessor product Quixil^®^/Crosseal™, Evicel^®^ does not contain the clot-stabilizing agent tranexamic acid (TA). Because preclinical in vivo studies showed that TA could cause seizures and direct effects on neuronal cells when applied to cortex and spinal cord [35–41], Quixil^®^/Crosseal™ was contraindicated in procedures where contact with CSF could occur [24]. In the current trial, most AEs in the nervous system disorder category were single events, except for headache, which occurred in circa one third of patients in each group. Seizures were noted in 1 Evicel^®^ subject and in 1 Sutures subject, who had prior histories of seizures.

Although small, the study population was a representative sample of the pediatric neurosurgery population. Intracranial tumor was the most frequent surgical indication, followed by epilepsy. The surgeries involved almost exclusively craniotomies, mostly with a supratentorial approach. These distributions resemble those of other large pediatric series [4, 21]. Infratentorial tumors are more frequent in children than in adults but occur mostly in the first decade of life [42, 43]. The low proportion of infratentorial approaches in this trial is likely due to the wide age range of subjects, half of them being 10 years or older. Moreover, the exclusion of children with hydrocephalus or CSF drainage resulted in the exclusion of patients with Chiari malformation, a pediatric neurosurgical indication that typically requires an infratentorial approach. Surgeries for epilepsy were all supratentorial, consistent with epilepsy surgery commonly being performed on the cerebral lobes [44].

This study presents certain limitations. The study size was small, and data analyses were descriptive. However, designed within the MHRA and EMA PIP frameworks, it is the first to prospectively investigate a dural sealant in the pediatric population, with a randomized controlled design and intra-operative efficacy endpoint. Exclusion of study subjects with major protocol deviations further reduced sample size. However, the PP and Safety sets maintained allocation ratios close to the intended 2:1 ratio, and sensitivity analyses showed consistent efficacy results with ratios of success proportions larger than 2. The exclusions from the PP set concerned 3 Evicel^®^ subjects and 1 Sutures subject with success, and 4 Sutures subjects with failure, placing the Evicel^®^ group at a disadvantage for efficacy analysis. Infratentorial procedures, known to confer a higher risk for CSF leak, were underrepresented. This is due to the relatively small sample size in this study and the fact that the proportion of these patients is a priori expected to be low [4, 21]. In the prior adult trial, however, the success rate of Evicel^®^ was significantly superior to that of sutured dural closure in the infratentorial as well as the supratentorial stratum [9], and, despite the low numbers, the current trial showed a similar trend.

In conclusion, the findings from this trial support the rationale that Evicel^®^ is safe and effective as an adjunct to primary sutured closure of the dura mater in the pediatric population and helps in preventing postoperative CSF leakage and associated surgical site complications. These results closely mirror those from a similar trial with Evicel^®^ conducted previously in an adult population [9].

### Electronic supplementary material

Below is the link to the electronic supplementary material.

Supplementary Material (DOCX 99.9 KB)

## Data Availability

All data generated or analyzed during this study are included in this published article and its Supplementary Material online, and at https://clinicaltrials.gov/ct2/show/study/NCT02309645.
