# Correction to: Safety and effectiveness of Evicel® fibrin sealant as an adjunct to sutured dural repair in children undergoing cranial neurosurgery

**DOI:** 10.1007/s00381-024-06503-8

**Published:** 2024-07-12

**Authors:** Gnanamurthy Sivakumar, Shailendra Magdum, Kristian Aquilina, Jothy Kandasamy, Vivek Josan, Bogdan Ilie, Ellie Barnett, Richard Kocharian, Benedetta Pettorini

**Affiliations:** 1 Leeds General Infirmary, Great George Street, Leeds, LS1 3EX UK; 2 John Radcliffe Hospital, Headley Way, Headington, Oxford, OX3 9DU UK; 3 Great Ormond Street Hospital, London, WC1N 3JH UK; 4 Royal Hospital for Sick Children, Sciennes Road, Edinburgh, EH9 1LF UK; 5 Royal Manchester Children’s Hospital, Oxford Road, Manchester, M13 9WL UK; 6 Ethicon, Inc., 1000 US-202 South, Raritan, NJ 08869 USA; 7 Ethicon, Inc., 8 Deer Park, Livingston, EH54 8AF UK; 8 Ethicon, Inc., 1000 US-202 South, Raritan, NJ 08869 USA; 9 Alder Hey Children’s Hospital, Eaton Road, Liverpool, L12 2AP UK

**Correction to: Child’s Nervous System**

https://doi.org/10.1007/s00381-024-06434-4

In Tables 1, 2, 3, 4, 5, 6 of this article, there are sub-entries that requires indentions for readers clarity. Below are the updated tables.

**Table 1:** Patient Characteristics (ITT Set). Medical history shows disorders that occurred in ≥10% of subjects.

	Evicel® (N=25)	Sutures (N=15)
Age (years), median (range)	10.0 (0.8,17.0)	10.0 (0.6,15.0)
Male/Female ratio, n (%)/n (%)	14 (56.0 %) / 11 (44.0%)	9 (60.0%) / 6 (40.0%)
BMI (kg/m^2^), median (range) ^a^	18.1 (13.4,33.8)	18.5 (14.0,23.7)
Medical History, n (%)		
Any previous surgery	8 (32.0%)	4 (26.7%)
Neoplasms	16 (64.0%)	11 (73.3%)
Nervous system disorders	14 (56.0%)	12 (80.0%)
Congenital/genetic disorders	9 (36.0%)	4 (26.7%)
Immune disorders	4 (16.0%)	0
Cardiac disorders	2 (8.0%)	2 (13.3%)
Infectious disorders	4 (16.0%)	0

^a^ Evicel® n=15, Sutures n=10

**Table 2:** Procedural Characteristics (ITT Set).

	Evicel® (N=25)	Sutures (N=15)
**Operative procedure**		
Procedure type, n subjects (% of total N)		
Craniotomy	24 (96.0%)	15 (100.0%)
Craniectomy	1 (4.0%)	0 (0.0%)
Approach, n subjects (% of total N)		
Infratentorial	4 (16.0%)	3 (20.0%)
Intracranial tumor	3 (12.0%)	3 (20.0%)
Chiari malformation	1 (4.0%)	0
Supratentorial	21 (84.0%)	12 (80.0%)
Intracranial tumor	13 (52.0%)	9 (60.0%)
Epilepsy	6 (24.0%)	1 (6.7%)
A-V malformation	2 (8.0%)	1 (6.7%)
Arachnoid cyst	0	1 (6.7%)
CSF leak before randomization, n subjects (% of total N)		
Spontaneous	14 (56.0%)	10 (66.7%)
After Valsalva Maneuver	11 (44.0%)	5 (33.3%)
Duration of surgery, min, median (range) ^a^	305 (123, 452)	288 (188, 675)
Time in operation room, min, median (range) ^a^	376 (160, 561)	361 (214, 778)
Postoperative hospital stay, nights, median (range)	5 (2, 25)	7 (2, 35)

^a^ Evicel® n=23, Sutures n=14.

**Table 3:** Treatment Parameters and Intra-operative Outcomes (Safety Set).

**Evicel**®		**N = 26**
Number of layers within each treatment, n subjects (% of total N = 26)		
*1*^*st*^* Treatment*	1 layer	18 (69.2%)
	2 layers	8 (30.8%)
*2*^*nd*^* Treatment*	1 layer	1 (3.9%)
	2 layers	2 (7.7%)
Intra-operative outcome following each treatment, n subjects (% of total N = 26)		
*1st Treatment*	No CSF Leak	23 (88.5%)
	CSF Leak	3 (11.5%)
*2nd Treatment*	No CSF Leak	0
	CSF Leak	3 (11.5%)
Rescue treatment, n subjects (%)		3 (11.5%)^a^

**Sutures**		**N = 14**
Number of additional sutures, median (range)		2.0 (1.0,12.0)
Additional treatment for durability, n subjects (%)		1 (7.1%) ^b^
Intra-operative outcome, n subjects (% of total N)		
	No CSF leak	5 (35.7%)
	CSF leak	9 (64.3%)
Rescue treatment, n subjects (% of total N)		9 (64.3%)^c^

^a^Infratentorial: DuraSeal® n = 1, Surgicel® + Duragen® + Duraguard® n = 1. Supratentorial: Tisseel® + Surgicel® n = 1

^b^Surgicel® Fibrillar

^c^Infratentorial: Pericranium n = 1, Adherus® + Duraguard® n = 1. Supratentorial: Tisseel® n = 2, Tisseel® + Surgicel® n = 1, Tisseel® + Duragen® n = 1, Tisseel® + Surgicel® + fascia n = 1, Surgicel® + Gelfoam® n = 1, Gelfoam® + Spongostan® n = 1

**Table 4:** Primary Efficacy Analysis and Sensitivity Analyses. Success was defined as the achievement of intra-operative watertight closure after completion of the randomized treatment, as assessed by provocative testing with Valsalva maneuver.

	Evicel® Success Rate % (n/N)	Sutures Success Rate % (n/N)	Estimated P_E_/P_S_	Farrington-Manning 95% CI for P_E_/P_S_
***Intention-to-treat set***				
Overall group	92.0% (23/25)	33.3% (5/15)	2.76	(1.53, 6.16)
Infratentorial	50.0% (2/4)	0.0% (0/3)		
Supratentorial	100.0% (21/21)	41.7% (5/12)		
***Per protocol set***				
Overall group	90.9% (20/22)	40.0% (4/10)	2.27	(1.27, 5.53)
***Safety set***				
Overall group	88.5% (23/26)	35.7% (5/14)	2.48	(1.39, 5.52)

P_E_/P_S_, Proportion successes in Evicel® group / Proportion successes in Sutures group

CI, Confidence Interval

**Table 5:** Adverse Events and Relatedness to Study Product (Determined for Evicel® Group Only) and Procedure (Safety Set).

	Evicel® (N=26)	Sutures (N=14)
**Individual AE, n (%)**		
Individual AEs	118	110
Related or possibly related to study product	0	N/A
Related or possibly related to study procedure	71 (60.2%)	73 (66.4%)
Individual SAEs	7	16 ^a^
Related or possibly related to study product	1 (3.8%) ^b^	N/A
Related or possibly related to study procedure	6 (85.7%) ^c^	14 (87.5%) ^d^
**Subjects with AE, n (%)**		
≥ 1 A*E*	22 (84.6%)	14 (100.0%)
≥ 1 Serious AE	5 (19.2%)	8 (57.1%)
≥ 1 Severe AE	2 (7.7%)	1 (7.1%)
≥ 1 AE related or possibly related to product	0	N/A
≥ 1 SAE related or possibly related to product	1 (3.8%) ^b^	N/A
≥ 1 AE related or possibly related to procedure	21 (80.8%)	12 (85.7%)
≥ 1 SAE related or possibly related to procedure	5 (19.2%)	7 (50.0%)

^a^ Partial seizure, neurofibromatosis and 14 other SAE as detailed in footnote (d)

^b^ Pseudomeningocele (Causality upgraded by Sponsor)

^c^ Diabetes insipidus, pyrexia, meningitis, medulloblastoma recurrence, convulsive seizure, hydrocephalus (due to malfunction existing CSF shunt),

^d^ Pseudomeningocele with iCSF leak (n=1), pseudomeningocele without iCSF leak (n=4), hematoma (n=2), vomiting (n=1), hemorrhagic cyst (n=1), shunt infection (n=1), pneumocephalus (n=1), transverse sinus thrombosis (n=1), hydrocephalus (due to malfunction existing CSF shunt) n=1, hydrocephalus (due to choroid plexus carcinoma and intraventricular blood collection) n=1.

**Table 6:** Surgical Site Complications Observed within 30 Days Postoperatively (Safety Set). All complications are also reported as AEs (Table 5).

Surgical site complications, n subjects (%)	Evicel® (N=26)	Sutures (N=1
≥ 1 surgical site complication	9 (34.6%)	8 (57.1%)
CSF leakage	1 (3.8%)	5 (35.7%)
Incisional CSF leakage	0	0
Pseudomeningocele	1 (3.8%) ^a^	4 (28.6 %) ^b^
Pseudomeningocele and incisional CSF leakage	0	1 (7.1%) ^c^
Infection	1 (3.8%)	1 (7.1%)
Hematoma	1 (3.8%)	1 (7.1%)
Other	6 (23.1%) ^d^	6 (42.9%) ^e^

^a^ No treatment needed.

^b^ Treated with CSF shunt (n=2), no treatment needed (n=2).

^c^ Treated with pressure bandage and re-suturing.

^d^ Itchy wound, soreness/numbness, hydrocephalus (due to malfunction existing CSF shunt), subgaleal collection, subgaleal swelling, weeping sutures.

^e^ Hydrocephalus (due to intraventricular blood collection), hydrocephalus (due to malfunction existing CSF shunt), non-occlusive sinus transversus thrombus, wound swelling, pneumocephalus, bruising.

The original article has been corrected.

