# Supplementary material for: Safety and effectiveness of Evicel® fibrin sealant as an adjunct to sutured dural repair in children undergoing cranial neurosurgery
Source: Childs Nerv Syst. 2024 May 10;40(9):2735–45. doi: 10.1007/s00381-024-06434-4 (PMC11322203; doi:10.1007/s00381-024-06434-4)
Supplement: Supplementary file 1 — Supplementary Material (DOCX 99.9 KB) [file 381_2024_6434_MOESM1_ESM.docx]

**Child’s Nervous System**

Safety and Effectiveness of EVICEL® Fibrin Sealant as an Adjunct to Sutured Dural Repair in Children undergoing Cranial Neurosurgery

**Supplementary Material**

Gnanamurthy Sivakumar*, Shailendra Magdum,

Kristian Aquilina, Jothy Kandasamy, Vivek Josan,

Bogdan Ilie, Ellie Barnett, Richard Kocharian, Benedetta Pettorini.

***Corresponding author:**

Gnanamurthy Sivakumar

Leeds General Infirmary, Great George Street, Leeds, LS1 3EX, UK

Telephone: +44 (113) 392 8567

Email: [gsivakumar1@nhs.net](mailto:gsivakumar1@nhs.net)

Table of contents

**Supplementary Material 1**. Inclusion and Exclusion Criteria Page S1

**Supplementary Material 2.** The quantitative use of Evicel® product (ITT set) Page S3

**Supplementary Material 1**. Inclusion and Exclusion Criteria.

Inclusion Criteria

Preoperative

1. Patient undergoing elective or urgent craniotomy/craniectomy for pathological processes in the posterior fossa (such as benign or malignant tumors, vascular malformation, and Chiari 1 malformations) or in the supratentorial region and who are demonstrated to have persistent CSF leakage following primary attempt at suture closure of the dural incision.

2. Administration of perioperative antibiotic prophylaxis.

3. Patients who are less than 18 years of age.

4. Patients who are able and willing to comply with the procedures required by the protocol.

5. The subject’s parent/legal guardian must be willing to give permission for the subject to participate in the trial and provide written informed consent for the subject. In addition, assent must be obtained from paediatric subjects who possess the intellectual and emotional ability to comprehend the concepts involved in the trial.

Intra-operative

1. Surgical wound classification Class I. Penetration of mastoid air cells during partial mastoidectomy is permitted.

2. The cuff of native dura along the craniotomy edge on each side is wide enough based on surgeon’s judgment to facilitate suturing and to allow for sufficient surface area for adherence of the investigational product.

Exclusion Criteria

Preoperative

1. Subjects with a dura lesion from a recent surgery that still has the potential for CSF leakage.

2. Conditions or treatments significantly compromising the immune system (such as AIDS).

3. Known hypersensitivity to the components (human fibrinogen, arginine hydrochloride, glycine, sodium chloride, sodium citrate, calcium chloride, human thrombin, human albumin, mannitol, and sodium acetate) of the investigational product.

4. Hydrocephalus (occlusive hydrocephalus is permitted when caused by posterior fossa pathology to be treated, i.e., hydrocephalus is due to blockage caused by a tumor to be removed).

5. Existing CSF (ventricular, etc.) drains, shunts, Cushing/Dandy cannulation, or burr holes which damage the dura.

6. Female subjects of childbearing potential with a positive urine or serum pregnancy test within 24 hours prior to surgery.

7. Female subjects who are breastfeeding, pregnant, or intend to become pregnant during the clinical study period.

8. Participation in another clinical trial with exposure to another investigational drug or device within 30 days prior to enrollment or expected during the study period.

9. Scheduled or foreseeable surgery within the follow-up period.

Intra-operative

1. Dura injury during craniotomy/craniectomy that cannot be eliminated by widening the craniotomy/craniectomy to recreate the native dura cuff.

2. Use of implants made of synthetic materials coming into direct contact with dura (e.g., PTFE patches, shunts, ventricular and subdural drains).

3. Planned use of dural patches after primary suture closure of the dura.

4. Placement of Gliadel Wafers.

5. Persistent signs of increased brain turgor.

6. Patient has a gap between durotomy edges of greater than 2 mm after primary dural closure.

7. Intersecting durotomy scars in the surgical path from a previous operation that cannot be completely removed by the planned dura resection.

8. Two or more separate dura defects, including defects from ventricular cannulation and ventricular-peritoneal shunting.

9. Major intra-operative complications that require resuscitation or deviation from the planned surgical procedure.

**Supplementary Material 2.** The quantitative use of Evicel® product (ITT set).

One Evicel® kit was used in 23 subjects (92.0%) and 2 kits in 2 subjects (8.0%), with a median (range) use of 4.0 (2.0, 8.0) mL of EVICEL® product per subject. Of a total of 27 applications, 1 (3.7%) was performed using the Spray, 25 using the drip (92.6%), and 1 using both (3.7%). The Airless spray tip was used in 1 (3.7%) application, the 4 cm Control Tip in 16 (59.3%) and the 6 cm Yellow Flexible Tip in 10 (37.0%) applications.
